# Antibiotic prescribing by age, sex, race, and ethnicity for patients admitted to the hospital with community-acquired bacterial pneumonia (CABP) in the *All of Us* database

**DOI:** 10.1017/cts.2023.567

**Published:** 2023-05-26

**Authors:** Corbyn M. Gilmore, Grace C. Lee, Susanne Schmidt, Christopher R. Frei

**Affiliations:** 1 Joe R. and Teresa Lozano Long School of Medicine and Graduate School of Biomedical Sciences, University of Texas Health San Antonio, San Antonio, TX, USA; 2 College of Pharmacy, The University of Texas at Austin, San Antonio, TX, USA; 3 South Texas Veterans Health Care System, San Antonio, TX, USA; 4 University Hospital, San Antonio, TX, USA; 5 School of Public Health, University of Texas Health Houston, San Antonio, TX, USA

**Keywords:** Antibiotics, race, ethnicity, older age, health disparities, epidemiology, clinical practice guidelines

## Abstract

**Purpose:**

To assess the proportion of inpatients who received guideline-concordant antibiotics for community-acquired bacterial pneumonia (CABP) in special populations of the *All of Us* database.

**Background:**

CABP contributes significantly to healthcare burden worldwide. The American Thoracic Society and Infectious Disease Society of America jointly published guidelines for the treatment of CABP. Guideline-concordant antibiotics for CABP are associated with better patient and cost outcomes.

**Methods:**

This was a retrospective cohort study of patients with pneumonia (*n* = 1608; SNOMED 233604007) from 10/1/2018 to 1/01/22 in the *All of Us* database. Cases were excluded for treatment setting other than inpatient, prior (within 90 days) pneumonia, receipt of intravenous antibiotics, respiratory isolation of methicillin-resistant *Staphylococcus aureus* (MRSA) or *Pseudomonas aeruginosa*, and/or other non-community-acquired types of pneumonia. Patients were grouped based on patient age, sex, race, and ethnicity. The proportion of patients on guideline-concordant therapy was compared within groups using chi-square statistics. Significant associations were assessed using multivariate logistic regression models.

**Results:**

A total of 1608 cases were included, and 45% of these patients received guideline-concordant antibiotics. Non-Hispanic White (NHW) patients vs. Black patients were associated with a 36% higher likelihood for receiving guideline-concordant antibiotics (adjusted OR, 1.36; 95% CI 1.02–1.81), whereas NHW vs. Hispanic patients were associated with a 34% lower likelihood for receiving guideline-concordant antibiotics (aOR 0.66; 0.48–0.91).

**Conclusion:**

Black patients with CABP in the *All of Us* database were less likely to receive guideline-concordant antibiotics, and Hispanic patients were more likely to receive guideline-concordant antibiotics, than NHW patients.

## Introduction

Pneumonia is a common type of lung infection in one or both lungs that can lead to serious complications including lung abscesses, respiratory failure, and sepsis [1]. Community-acquired bacterial pneumonia (CABP) is pneumonia caused by a bacterial pathogen – usually *Streptococcus pneumoniae* – in a community setting [1]. In 2019, influenza and pneumonia were the ninth leading cause of death in the USA with 49,783 deaths [2]. Annually, over one million hospitalizations and 540,000 emergency department visits in the USA are attributed to pneumonia [3]. For patients aged 65 years and older, rates of hospitalized CABP averaged 1899 per 100,000 person-years (PY) [3]. For patients younger than 65 years of age, rates of hospitalized CABP averaged 285 per 100,000 PYs [3]. Patients with CABP also create a substantial burden on hospital resources. For patients admitted to the hospital with CABP, mortality during hospitalization exceeds 6%, and the cost of treatment is approximately $17,736 per patient, with a hospital length of stay (LOS) averaging 5.7 days [4,5].

To decrease the burden associated with CABP, the American Thoracic Society (ATS) and the Infectious Disease Society of America (IDSA) jointly published guidelines for the management of CABP in 2019 [6]. These recommendations were developed by a multidisciplinary panel conducting systematic reviews of relevant literature and clinical trials [6]. Guideline-concordant antibiotic therapy has been associated with shorter time to clinical stability [7], shorter time to switch therapy [7], reduced hospital LOS [7,8], and improved patient survival in patients with CABP admitted to the hospital [7].

Investigations into racial and/or ethnic disparities in prescribing patterns of guideline-concordant antibiotic therapy for CABP have been sparse since the recommendations were published in 2019. In prior reports, both in the general inpatient ward and intensive care settings, Black and Non-Hispanic White (NHW) patients have been observed to be equally as likely to receive ATS/IDSA guideline-concordant antibiotic therapy [9,10]. Additionally, 30-day mortality was similar between groups in the general inpatient ward but was found to be lower for Black patients vs. NHW patients when admitted to the intensive care unit (ICU) [9]. Further, Hispanic and Asian patients had lower mortality than NHW or Black patients [11,12]. However, Black and Hispanic patients were less likely to receive timely initiation (within four hours) of antibiotic therapy [9–11], appropriate vaccination (pneumococcal and influenza) [10,14], and smoking cessation counseling [14]. These differences are seen more prominently between different hospitals than within the same hospital [9,10,14]. There has been little evidence of race- or ethnicity-based differences in guideline-concordant prescribing for the management of CABP [9,11]. Of note, guideline-concordant antibiotic prescribing for patients admitted to the hospital with CABP has not yet been evaluated in the *All of Us* cohort.

The *All of Us* Research Program is an effort led by the National Institutes of Health (NIH) to increase the rate of health-related research discoveries, further develop precision medicine, and analyze the impact of environment, lifestyle, genetics, and other determinants of health on disease and health [15]. To accomplish this, the *All of Us* Research Program is working to create a diverse health database that includes data from groups traditionally underrepresented in research by enrolling at least 1,000,000 people from the USA over the span of 10 years [15]. To date, 590,000+ participants have consented to be enrolled and 411,000+ participants have completed the first steps of the program [16]. The first steps of the program provide baseline data for each participant, and they include consenting, providing physical measurements, the donation of at least one biospecimen to the *All of Us* biobank, agreement to share electronic health records, and completion of the first three surveys [16]. The data are publicly available and open-sourced with access being stratified into three tiers to protect participant privacy (Public, Registered, and Controlled Tier) [17]. Data are collected through participant-provided information (PPI), electronic health records, biospecimens, and digital health devices (wearable sensors, apps, mobile phones, etc.) [15,16].

This research uses newly available *All of Us* data to examine guideline-concordant treatment of CABP. In this study, we identify whether disparities exist in guideline-concordant antibiotic prescribing among patients admitted to the hospital for CABP in the *All of Us* database by age-, race-, sex-, and ethnicity-based subgroups.

## Methods

### Regulatory and Ethics

All data collection, analysis, and dissemination were conducted in accordance with the *All of Us* Data User Code of Conduct and all policies therein. Institutional Review Board approval was obtained from University of Texas Health Science Center at San Antonio Institutional Review Board (IRB) and Office of Clinical Research (OCR) before beginning the study (Protocol number: 20220682EX).

### Data Source and Study Population

We identified all adult patients with a diagnosis of pneumonia (SNOMED 233604007), or one of its descendent concepts (*n* = 21), after October 1, 2018, in the *All of Us* database using version 6 of the Curated Data Repository (CDR). Version 6 of the CDR (released June 2022) includes data collected between October 1, 2018, and January 1, 2022. The date used for screening is one year before the publication of the 2019 ATS/IDSA guidelines. This is done to account for the “date shifting” found in the *All of* Us database. This shift translates to all dates associated with a participant shifted back 1–365 days, but the shift is constant for each participant to preserve temporality of events [18]. The *All of Us* database collects both gender identity and sex assigned at birth using “The Basics” survey, which is PPI [19]. For this study, sex was determined using data from the sex assigned at birth question.

#### Inclusion and Exclusion Criteria

Patients included in this study were those who had a diagnosis of pneumonia present in their record and were prescribed antibiotics during the same encounter as the pneumonia diagnosis.

Patients were excluded for the following:

- Patient must not have had a previous case of pneumonia less than 90 days prior to current case. For patients with multiple cases on record, there must have been an uninterrupted span of at least 90 days. If there was not an uninterrupted span of 90 days between cases, then the earliest visit was counted.
- Patient must not have had parenteral antibiotics prescribed during an inpatient visit in the 90 days before the pneumonia encounter.
- Patient must not have had a prior isolation of methicillin-resistant *Staphylococcus aureus* (MRSA).
- Patient must not have had a prior isolation of *Pseudomonas aeruginosa*.
- Pneumonia visit must have had a designation of inpatient or outpatient visit.
- Encounter must not have been an ICU visit.
- Patient must not have lived in a nursing facility in the 90 days before the pneumonia case.
- The pneumonia case must not have been due to chronic, aspiration, hospital acquired, or ventilator acquired pneumonia.

#### Data Collection

Information was extracted from the *All of Us* database using the cohort builder tool and analyzed using the Jupyter notebook analysis environment inside of *All of Us* Researcher Workbench. Information was extracted using the cohort builder tool, concept sets for pneumonia, demographics, and antibiotic treatment received, and dataset builder tool. All tools used were found in the *All of Us* Researcher Workbench. The data access tier used was the Registered Tier, which contains individual-level data including electronic health records (EHRs), wearables, survey responses, and physical measurements.

### Study Design

This is a retrospective cohort study of patients admitted to the hospital with pneumonia after October 1, 2018, through January 1, 2022, in the *All of Us* database (*n* = 1608). Patients were stratified into groups based on whether they received guideline-concordant or guideline-discordant antibiotic therapy as defined by the 2019 ATS/IDSA guidelines at any time during their admission [6]. They were then further stratified into subgroups based on patient age [≤65 years (*n* = 994) vs. 65+ years (*n* = 614)], sex [female (*n* = 807) vs. male (*n* = 780)], race [Black (*n* = 560) vs. NHW (*n* = 592)], and ethnicity [Hispanic (*n* = 336) vs. NHW (*n* = 592)]. For the race subgroup, patients who did not identify as NHW or Black or did not indicate their race were categorized as Other. For the ethnicity subgroup, patient who did not identify as NHW or Hispanic or did not indicate their ethnicity were classified as Other. Percent of subgroups who received guideline-concordant antibiotic therapy was then compared using chi-square analysis. Then, when there were statistically significant differences (*p* < 0.05) between the subgroups, multivariate logistic regression models were created with subgroup as the independent variable, guideline-concordant therapy as the dependent variable, and divergent (significantly different) baseline characteristics as covariates. Specific medications prescribed were then cataloged, and percentage of subgroups who received the specific medications were compared using chi-square analysis.

### Study Variables and End Points

#### Guideline-Concordant and -Discordant Definitions

The 2019 guidelines for empiric antibiotic treatment of bacterial pneumonia, as defined by the ATS/IDSA, were used to subset patients into two groups based on the antibiotics prescribed during the pneumonia visit. Patients who received β-Lactam (e.g., ampicillin + sulbactam, cefotaxime, ceftriaxone, or ceftaroline) and macrolide (azithromycin or clarithromycin) combination therapy, β-Lactam and doxycycline combination therapy, or respiratory fluoroquinolone (levofloxacin or moxifloxacin) monotherapy were considered to have been prescribed “guideline-concordant” antibiotics [6]. We used the 2019 ATS/IDSA guidelines as our reference for what constitutes guideline-concordant antibiotic therapy, but these antibiotic recommendations have been consistent for several versions of the guidelines – well before 2019 – so all patient visits in this study occurred well after adoption of these antibiotic recommendations into evidence-based guidelines. Patients who received other regimens were considered to have received “guideline-discordant” antibiotics [6].

#### Outcomes

The primary outcome of this study was the receipt of guideline-concordant antibiotic therapy as defined by the 2019 ATS/IDSA guidelines for the management of patients admitted to the hospital for CABP.

### Statistical Analysis

Jupyter notebook inside the *All of Us* Researcher Workbench was the analysis environment used for all statistical comparisons. The alpha level for significance was 0.05. Patient age, the only numeric variable, was not normally distributed, so we reported median and interquartile range and used the Wilcoxon rank-sum test to compare patient age between groups. Dichotomous variables like baseline characteristics and prescribing practices based on age, sex, race, and ethnicity were compared using chi-square or Fischer’s exact tests. Odds ratios and 95% confidence intervals for the receipt of guideline-concordant antibiotic therapy were calculated as well. Then, for subgroups where there was a statistically significant difference in antibiotic prescribing between groups, multivariate logistic regression models were created with subgroup as the independent variable, guideline-concordant therapy as the dependent variable, and divergent baseline characteristics as covariates. Patients who chose skip (*n* = 84), prefer not to answer (*n* = 63), or don’t know (*n* = 10) for any of the categories were excluded from logistic regression of race and guideline-concordant prescribing (note: categories are not mutually exclusive). Patients who chose skip (*n* = 60), prefer not to answer (*n* = 23), or don’t know (*n* = 15) for any of the categories were excluded from logistic regression of ethnicity and guideline-concordant Prescribing (note: categories are not mutually exclusive).

## Results

**Figure 1.**
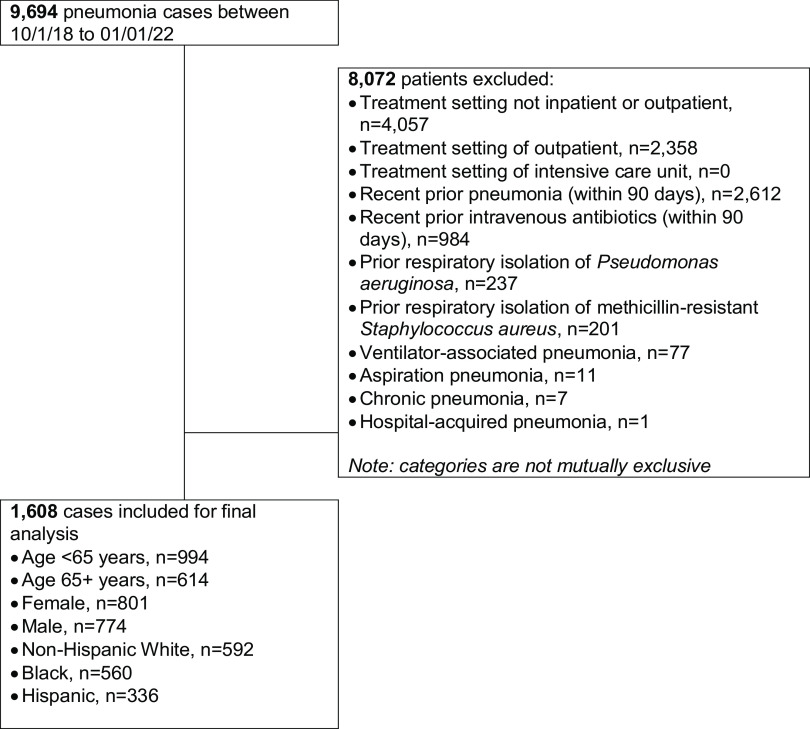
Study inclusion flow chart.

A total of 1608 patients with CABP met study inclusion/exclusion criteria (Fig. 1) and 45% (729/1608) received guideline-concordant antibiotic therapy. The guideline-concordant group was composed of patients treated with β-Lactam plus a macrolide (68%, 496/729), β-Lactam plus doxycycline (18%, 131/729), and fluoroquinolone monotherapy (33%, 241/729).

### Patient Demographics

Table 1 represents demographics for patients admitted to the hospital with CABP and treated with guideline-concordant and -discordant antibiotic therapy in the *All of Us* database after October 1, 2018. There were significant differences in the percentage of patients prescribed guideline-concordant antibiotics between Black and NHW patients (*p* = 0.01) and between Hispanic and NHW patients (*p* = 0.02). The two groups were statistically similar regarding age, sex, smoking status, electronic smoking status, alcohol use, employment status, marital status, and insurance status.

**Table 1.** Patient baseline characteristics by guideline-concordant and -discordant antibiotics^*,**^

Characteristic, % (except for age)	Guideline-concordant, *n* = 729	Guideline-discordant, *n* = 879	*P*-value
Age, median (interquartile range)	62 (53–71)	62 (53–70)	0.68
Race	–	–	***<0.01***
Non-Hispanic White	277 (38%)	315 (36%)	–
Black	216 (30%)	344 (39%)	–
Other or Not Indicated	299 (32%)	220 (25%)	–
Ethnicity	–	–	***<0.01***
Not Hispanic or Latino	521 (72%)	690 (79%)	
Hispanic	184 (25%)	152 (17%)	–
Other or Not Indicated	24 (3%)	37(4%)	–
Sex	–	–	0.19
Female	373 (51%)	428 (49%)	–
Male	345 (47%)	429 (49%)	–
Other or Not Indicated	≤20	22 (3%)	–
Smoking status	–	–	0.32
100 Cigarettes Lifetime: No	331 (45%)	365 (42%)	–
100 Cigarettes Lifetime: Yes	373 (51%)	488 (56%)	–
Not Indicated	25 (3%)	26 (3%)	–
Electronic Smoke Participant	–	–	0.77
Electronic Smoke Participant: No	610 (84%)	752 (86%)	–
Electronic Smoke Participant: Yes	92 (13%)	95 (11%)	–
Not Indicated	27 (4%)	30(4%)	–
Alcohol Use	–	–	***0.05***
Alcohol Use: No	105 (14%)	138 (16%)	–
Alcohol Use: Yes	605 (83%)	716 (82%)	–
Not Indicated	≤20	≤20	–
Employment Status	–	–	0.67
Employed for wages or self-employed	160 (22%)	186 (21%)	–
Not currently employed for wages	544 (75%)	652 (74%)	–
Not Indicated	25 (4%)	41 (5%)	–
Marital Status	–	–	0.19
Divorced	153 (17%)	153 (17%)	–
Living with Partner	33 (5%)	40 (5%)	–
Married	256 (35%)	285 (32%)	–
Never Married	198 (27%)	237 (27%)	–
Separated	41 (6%)	42 (5%)	–
Widowed	66 (9%)	82 (9%)	–
Not Indicated	≤20	40 (5%)	–
Insurance Status	–	–	0.48
Health Insurance: No	27 (4%)	29 (3%)	–
Health Insurance: Yes	683 (94%)	821 (93%)	–
Not Indicated	≤20	29 (3%)	–

* Bold italics indicate statistically significant findings.

** Counts of less than or equal to 20 are displayed as ≤20 to comply with the *All of Us* data and statistic dissemination policy [33].

Table 2 represents patient baseline characteristics by race and ethnicity. There were significant differences in age (years; *p* < 0.01), age (≤65 years vs. >65 years; *p* < 0.01) electronic smoking status (*p* = 0.03), alcohol use (*p* < 0.01), employment status (*p* < 0.01), marital status (*p* < 0.01), and insurance status between NHW and Black patients (*p* = 0.01). Between NHW and Hispanic patients, there were significant differences in age (*p* < 0.01), age (≤65 years vs. >65 years; *p* < 0.01), smoking status (*p* < 0.01), electronic smoking status (*p* = 0.02), alcohol use (*p* < 0.01), and marital status (*p* < 0.01).

**Table 2.** Patient baseline characteristics by race and ethnicity^*,**^

Characteristic, % (except for age)	NHW, *n* = 592	Black, *n* = 560	P-value (Non-Hispanic White vs. Black)	Hispanic, *n* = 336	*P*-value (Non-Hispanic White vs. Hispanic)
Age (years), median (interquartile range)	67 (57–74)	59 (52–66)	***<0.01***	60 (50–69)	***<0.01***
≤65 years	47%	73%	***<0.01***	69%	***<0.01***
>65 years	53%	27%	–	31%	–
Sex	–	–	0.45	–	0.65
Female	50%	50%	–	51%	–
Male	48%	49%	–	48%	–
Other or Not Indicated	≤20	≤20	–	≤20	–
Smoking status	–	–	0.20	–	***<0.01***
100 Cigarettes Lifetime: No	41%	36%	–	58%	–
100 Cigarettes Lifetime: Yes	56%	62%	–	39%	–
Not Indicated	≤20	≤20	–	≤20	–
Electronic Smoke Participant	–	–	***0.03***	–	***0.02***
Electronic Smoke Participant: No	85%	83%	–	90%	–
Electronic Smoke Participant: Yes	13%	12%	–	7%	–
Not Indicated	≤20	5%	–	≤20	–
Alcohol Use	–	–	***<0.01***	–	***<0.01***
Alcohol Use: No	5%	17%	–	30%	–
Alcohol Use: Yes	94%	79%	–	69%	–
Not Indicated	≤20	4%	–	≤20	–
Employment Status	–	–	***<0.01***	–	0.11
Employed for wages or self-employed	27%	15%	–	21%	–
Not currently employed for wages	71%	81%	–	76%	–
Not Indicated	≤20	4%	–	≤20	–
Marital Status	–	–	***<0.01***	–	***<0.01***
Divorced	15%	19%	–	19%	–
Living with Partner	≤20	6%	–	5%	–
Married	52%	13%	–	38%	–
Never Married	16%	43%	–	20%	–
Separated	≤20	7%	–	9%	–
Widowed	12%	8%	–	8%	–
Not Indicated	≤20	5%	–	≤20	–
Insurance Status	–	–	***0.01***	–	0.09
Health Insurance: No	≤20	4%	–	≤20	–
Health Insurance: Yes	96%	94%	–	93%	–

* Bold italics indicate statistically significant findings.

** Counts of less than or equal to 20 are displayed as ≤20 to comply with the *All of Us* data and statistic dissemination policy [33].

### Antibiotic Prescribing Patterns and Health Disparities

Table 3 represents guideline-concordant prescribing rates by age, sex, race, and ethnicity. Forty-seven percent of NHW patients (277/592), 38% of Black patients (216/560), and 54% of Hispanic patients (184/336) received guideline-concordant antibiotic therapy. There were no other significant differences based on race or ethnicity in medication choice for the guideline-concordant group.

**Table 3.** Guideline-concordant antibiotic prescribing by age, sex, race, and ethnicity^*,**^

Characteristic	Guideline-concordant, *n* = 729	Guideline-discordant, *n* = 879	OR	95% CI	*P*-value
Age	–	–	1.04	0.85–1.27	0.73
≤65 years	62%	61%	–	–	–
>65 years	38%	39%	–	–	–
Sex	–	–	–	–	–
Female	51%	49%	0.92	0.76–1.13	0.43
Male	47%	49%	–	–	–
Race	–	–	–	–	–
Non-Hispanic White	38%	36%	***1.4***	***1.17–1.77***	***0.01***
Black	30%	39%	–	–	–
Ethnicity	–	–	–	–	–
Non-Hispanic White	38%	36%	***0.73***	***0.56–0.96***	***0.02***
Hispanic	25%	17%	–	–	–

* Bold italics indicate statistically significant findings.

** Counts of less than or equal to 20 are displayed as ≤20 to comply with the *All of Us* data and statistic dissemination policy [33].

Table 4 represents a breakdown of the medications prescribed for patients admitted to the hospital with CABP in the guideline-concordant group. Levofloxacin accounted for all fluoroquinolone use except for a subset of ≤20 patients who received moxifloxacin. Ceftriaxone was the only β-Lactam prescribed except for a subset of ≤20 patients who received ampicillin/sulbactam in combination with a macrolide. Azithromycin accounted for all macrolide use except for a subset of ≤20 patients who received clarithromycin. There were significant differences in prescribing rates of β-Lactams, specifically ceftriaxone, between Hispanic and NHW patients (*p* < 0.01). There were no other significant differences based on race or ethnicity in medication choice for the guideline-concordant group. Please refer to Supplemental Table 1 for a list of the most common guideline-discordant medications.

**Table 4.** Guideline-concordant antibiotics by race and ethnicity^*,**^

Antibiotic	NHW, *n* = 277	Black, *n* = 216	*P*-value (Black vs. Non-Hispanic White)	Hispanic, *n* = 184	*P*-value (Hispanic vs. Non-Hispanic White)
β-Lactam	72%	75%	0.51	84%	***<0.01***
Ampicillin-Sulbactam	≤20	≤20	–	≤20	–
Cefotaxime	0	0	–	0	–
Ceftriaxone	72%	75%	0.46	84%	***<0.01***
Ceftaroline	0	0	–	0	–
Macrolide	63%	70%	0.08	70%	0.10
Azithromycin	63%	70%	0.08	70%	0.10
Clarithromycin	0	≤20	–	0	–
Fluoroquinolone	35%	39%	0.36	27%	0.07
Levofloxacin	31%	37%	0.16	26%	0.28
Moxifloxacin	≤20	≤20	0.52	≤20	0.10
Doxycycline	19%	25%	0.13	20%	0.77
β-Lactam + Macrolide	63%	70%	0.08	70%	0.10
β-Lactam + Doxycycline	16%	21%	0.17	19%	0.33

* Bold italics indicate statistically significant findings.

** Counts of less than or equal to 20 are displayed as ≤20 to comply with the *All of Us* data and statistic dissemination policy [33].

Table 5 represents the multivariate logistic regression analysis of guideline-concordant antibiotic prescribing and race. NHW vs. Black patients were associated with a 36% higher likelihood for receiving guideline-concordant treatment (adjusted OR, 1.36; 95% CI 1.02–1.81); age strata (≤65 vs. >65), electronic smoking use, alcohol use, employment status, marital status, and insurance status were used as covariates. Table 6 represents the multivariate logistic regression analysis of guideline-concordant antibiotic prescribing and ethnicity. NHW vs. Hispanic patients were associated with a 34% lower likelihood of receiving guideline-concordant antibiotics (aOR 0.66; 0.48–0.91); age strata [≤65 vs. >65], smoking status, electronic smoking use, alcohol use, employment status, and marital status were used as covariates.

**Table 5.** Logistic regression analysis of race and guideline-concordant antibiotic prescribing^*,**,***^

Characteristics	Estimate	OR	95% CI	*P*-value
Age (>65 vs. ≤65 years)	−0.12 ± 0.14	0.89	0.68–1.17	0.40
Race (Non-Hispanic White vs. Black)	***0.31 ± 0.15***	***1.36***	***1.02–1.81***	***0.04***
Electronic Smoke Participant (Yes vs. No)	0.23 ± 0.19	1.26	0.86–1.82	0.23
Alcohol Use (Yes vs. No)	0.20 ± 0.22	1.23	0.80–1.90	0.35
Employment Status (No vs. Yes)	0.03 ± 0.16	1.03	0.76–1.41	0.84
Marital Status (Yes vs. No)	0.03 ± 0.15	1.03	0.77–1.38	0.85
Insurance Status (Yes vs. No)	−0.26 ± 0.41	0.77	0.34–1.72	0.52

* Bold italics indicate statistically significant findings.

** Patients who chose Skip (*n* = 84), Prefer not to answer (*n* = 63), or Don't Know (*n* = 10) for any of the categories were excluded (total *n* = 124) from logistic regression of Race and Guideline-Concordant Prescribing. (Note: categories are not mutually exclusive).

*** The second variable listed is the reference group used in this model.

**Table 6.** Logistic regression analysis of ethnicity and guideline-concordant antibiotic prescribing^*,**,***^

Characteristics	Estimate	OR	95%CI	*P*-Value
Age (>65 vs. ≤65 years)	0.21 ± 0.15	1.23	0.92–1.66	0.17
Ethnicity (Non-Hispanic White vs. Hispanic)	***0.42 ± 0.16***	***0.66***	***0.48–0.91***	***0.01***
Smoking status (Yes vs. No)	−***0.43 ± 0.15***	***0.65***	***0.48–0.88***	***<0.01***
Electronic Smoke Participant (Yes vs. No)	0.45 ± 0.24	1.57	0.99–2.51	0.06
Alcohol Use (Yes vs. No)	0.44 ± 0.23	1.55	0.996–2.41	0.053
Employment Status (No vs. Yes)	0.03 ± 0.17	1.03	0.74–1.44	0.85
Marital Status (Yes vs. No)	−0.02 ± 0.14	0.98	0.74–1.30	0.90

* Bold italics indicate statistically significant findings.

** Patients who chose Skip (*n* = 60), Prefer not to answer (*n* = 23), or Don't Know (*n* = 15) for any of the categories were excluded (total *n* = 72) from logistic regression of Ethnicity and Guideline-Concordant Prescribing. (Note: categories are not mutually exclusive).

*** The second variable listed is the reference group used in this model.

## Discussion

This study evaluated antibiotic prescribing for patients admitted to the hospital with CABP in the *All of Us* database. Previous studies have demonstrated that guideline-concordant antibiotic therapy is associated with improved clinical outcome for patients admitted to the hospital with CABP [6,7,20,21]. Improved clinical outcomes include decreased LOS, shorter time to switch therapy, decreased mortality, and shorter time to clinical stability [6–8,20,21]. Additionally, multiple studies have shown that guideline-concordant antibiotic therapy has been associated with lower total healthcare costs for ward patients hospitalized with CABP [8,22–24]. Identifying potential variation and disparity is the first step in correcting them and therefore improving patient outcomes.

Less than half (46%, 729/1608) of patients admitted to the hospital for CABP in the *All of Us* database were prescribed guideline-concordant antibiotic therapy. These patient visits took place in October 2018 through January 2022, and as such, the treating physician would have access to previous iterations of the guidelines [25]. One of the most significant changes between versions is the discontinuation of use of healthcare-associated pneumonia as a pneumonia category [6,21]. This was done to shift emphasis to local epidemiology, previously validated risk factors, and antibiotic stewardship [6,21]. In addition to this change, further evidence was gathered in favor of β-Lactam + macrolide combination therapy and fluoroquinolone monotherapy over β-Lactam monotherapy for the management of CABP [6,21]. And finally, further evidence has strengthened the recommendation for β-Lactam + macrolide combination therapy over fluoroquinolone monotherapy, although both are still recommended [6]. This is due in part to the potential symptoms of fluoroquinolone exposure, which include neuropathy, aortic ruptures and tears, tendinopathy, and *C. difficile* infection [21,26,27]. Individuals at higher risk for these complications include those with hypertension, a history of circulatory blockages or aneurisms, select genetic disorders, and the elderly [21,26–28]. Efficacy is similar between the two regimens; however, some studies have demonstrated that fluoroquinolone monotherapy leads to a reduces hospital LOS [9,28–31]. β-Lactam plus macrolide combination therapy was the most prescribed guideline-concordant therapy (68%, 496/729), with ceftriaxone as the β-Lactam and azithromycin as the macrolide.

While only half of CABP cases received guideline-concordant treatment, the guideline-concordant and -discordant groups were similar in their sociodemographic characteristics except in race, ethnicity, and alcohol use. Across race and ethnicity-based subgroups, NHW patients were significantly different from Black and Hispanic patients regarding age, electronic smoking status, alcohol use, employment status, and marital status. The proportion of NHW patients older than 65 years was significantly higher than both the Black and Hispanic patients. The cause for the difference in guideline-concordant prescribing based on race and ethnicity is unclear; however, process variations between hospitals may be a contributing factor [11,14]. Previous studies have identified other care gaps related to inpatient management of CABP for Black patients such as increased time to initiation of antibiotic therapy [10,11,13,14], decreased vaccination counseling [10,14], and decreased smoking cessation counseling [10]. Further research into the clinical decision processes and workflows surrounding the treatment of CABP is needed reduce the barriers associated with guideline-concordant antibiotic prescription. Additionally, cultural competency/proficiency/humility training of healthcare workers might help mitigate these differences in prescribing practices. Further optimization of these processes could yield improved compliance with guidelines, patient outcomes, and cost and reduce CABP-associated hospital burden.

Because of its observational nature, this study has potential limitations. Investigators were unable to account for all risk factors for MRSA and *P. aeruginosa* infection due to lack of local epidemiological data. Additionally, investigators could not account for institution-level and individual variations that go into the decision-making process for antibiotic selection, including allergies to medication. This study has potential selection bias as patients were not randomized to treatment arms. The *All of Us* database does not currently contain enough patients (i.e., inadequate sample size) to investigate associations between antibiotic prescribing and health outcomes but does contain valuable information on prescribing patterns for various special populations. We can infer, based on previous research from Frei *et al.* [7,8], that patient populations that receive less guideline-concordant antibiotics are also less likely to experience positive health outcomes. Finally, there were no pneumonia patients from the ICU setting in the version of the *All of Us* database we used for this study. Even if there had been, the CABP guidelines recommend different antibiotics for patients admitted to the hospital ward and ICU, so we would have had to stratify patients by setting before determining if the antibiotics received were guideline-concordant or -discordant. Despite these limitations, the present work provides valuable information for the management of inpatients with CABP from a database including patients underrepresented in research.

## Conclusions

In the *All of Us* database, Black patients admitted to the hospital with CABP were less likely to receive guideline-concordant antibiotic therapy when compared with NHW patients. In contrast, Hispanic patients admitted to the hospital with CABP were more likely to receive guideline-concordant antibiotic therapy than NHW patients. Identification of health disparities, such as differences in evidence-based antibiotic prescribing by race and ethnicity, are an important first step in combating health disparities.

## Supplementary material

For supplementary material accompanying this paper visit https://doi.org/10.1017/cts.2023.567. click here to view supplementary material
