# Supplementary material for: Antibiotic prescribing by age, sex, race, and ethnicity for patients admitted to the hospital with community-acquired bacterial pneumonia (CABP) in the All of Us database
Source: J Clin Transl Sci. 2023 May 26;7(1):e132. doi: 10.1017/cts.2023.567 (PMC10308426; doi:10.1017/cts.2023.567)
Supplement: Supplementary file 1 [file S2059866123005678sup001.docx]

**Supplemental Table 1.** Guideline-Discordant Antibiotics, by Race and Ethnicity

| Antibiotic | Non-Hispanic White n=320 | Black n=342 | P-value | Hispanic n=152 | P-value |
| --- | --- | --- | --- | --- | --- |
| β-Lactam | ***20%*** | ***12%*** | ***0.01*** | ***38%*** | ***<0.01*** |
| Macrolide | 32% | 38% | 0.17 | ***18%*** | ***<0.01*** |
| Azithromycin | 33% | 38% | 0.12 | ***18%*** | ***<0.01*** |
| Cephalosporin | ***10%*** | ***≤20*** | ***<0.01*** | ***≤20*** | ***0.01*** |
| Ceftriaxone | ***18%*** | ***12%*** | ***0.03*** | ***35%*** | ***<0.01*** |
| Cefpodoxime | ***7%*** | ***<20*** | ***<0.01*** | ***≤20*** | ***0.02*** |
| Cefepime | ***21%*** | ***13%*** | ***0.01*** | ***29%*** | ***0.05*** |
| Cefazolin | ***13%*** | ***7%*** | ***<0.01*** | ≤20 | 0.92 |
| Cedinir | ***≤20*** | ***9%*** | ***<0.01*** | ≤20 | 0.22 |
| Ceftazidime | ≤20 | ≤20 | 0.13 | ≤20 | 0.31 |
| Cefuroxime | ***≤20*** | ***≤20*** | ***0.02*** | ≤20 | 0.11 |
| Cephalexin | ≤20 | ≤20 | 0.16 | ≤20 | 0.48 |
| Fluoroquinolones | ≤20 | 9% | 0.13 | ***≤20*** | ***0.04*** |
| Levofloxacin | ≤20 | 7% | 0.48 | ***≤20*** | ***0.05*** |
| Moxifloxacin | ≤20 | ≤20 | 0.07 | ≤20 | 0.59 |
| Fluoroquinolones and  β-Lactam | ≤20 | ≤20 | 0.70 | ≤20 | 0.08 |
| Fluoroquinolones and  Macrolide | ***<20*** | ***6%*** | ***0.03*** | ≤20 | 0.35 |
| **Other** | - | - | - | - | - |
| Amoxicillin | 16% | 13% | 0.31 | ≤20 | ***0.02*** |
| Aztreonam | ***≤20*** | ***≤20*** | ***0.03*** | ≤20 | 0.52 |
| Bacitracin | ≤20 | ≤20 | 0.38 | ***≤20*** | ***0.04*** |
| Ciprofloxacin | ≤20 | ≤20 | 0.23 | ≤20 | 0.72 |
| Clavulanate | ***12%*** | ***≤20*** | ***<0.01*** | ***≤20*** | ***0.03*** |
| Doxycycline | ***11%*** | ***20%*** | ***<0.01*** | **≤20** | 0.62 |
| Linezolid | ≤20 | ≤20 | 0.74 | **≤20** | 0.40 |
| Fluconazole | ≤20 | ≤20 | 0.47 | ≤20 | 0.42 |
| Metronidazole | 12% | 13% | 0.62 | ≤20 | 0.99 |
| Meropenem | ≤20 | ≤20 | 0.07 | ≤20 | 0.31 |
| Piperacillin-Tazobactam | ***18%*** | ***7%*** | ***<0.01*** | ***33%*** | ***>0.01*** |
| Sulfamethoxazole | 13% | 11% | 0.66 | ≤20 | 0.99 |
| Trimethoprim | 11% | 8% | 0.15 | ≤20 | 085 |
| Vancomycin | ***51%*** | ***35%*** | ***<0.01*** | 46% | 0.35 |

* Bold italics indicates statistically significant findings.
